# Topic Analysis of UK Fitness to Practise Cases: What Lessons Can Be Learnt?

**DOI:** 10.3390/pharmacy7030130

**Published:** 2019-09-04

**Authors:** Alan Hanna, Lezley-Anne Hanna

**Affiliations:** 1 Queen’s Management School, Queen’s University Belfast, University Rd, Belfast BT7 1NN, UK; 2 School of Pharmacy, Queen’s University Belfast, University Rd, Belfast BT7 1NN, UK

**Keywords:** dental, fitness to practice, machine learning, medical, non-negative matrix factorization, nursing, pharmacy, text mining, topic analysis

## Abstract

**Background**: Fitness to practise (FtP) impairment (failure of a healthcare professional to demonstrate skills, knowledge, character and/or health required for their job) can compromise patient safety, the profession’s reputation, and an individual’s career. In the United Kingdom (UK), various healthcare professionals’ FtP cases (documents about the panel hearing(s) and outcome(s) relating to the alleged FtP impairment) are publicly available, yet reviewing these to learn lessons may be time-consuming given the number of cases across the professions and amount of text in each. We aimed to demonstrate how machine learning facilitated the examination of such cases (at uni- and multi-professional level), involving UK dental, medical, nursing and pharmacy professionals. **Methods**: Cases dating from August 2017 to June 2019 were downloaded (577 dental, 481 medical, 2199 nursing and 63 pharmacy) and converted to text files. A topic analysis method (non-negative matrix factorization; machine learning) was employed for data analysis. **Results**: Identified topics were criminal offences; dishonesty (fraud and theft); drug possession/supply; English language; indemnity insurance; patient care (including incompetence) and personal behavior (aggression, sexual conduct and substance misuse). The most frequently identified topic for dental, medical and nursing professions was patient care whereas for pharmacy, it was criminal offences. **Conclusions**: While commonalities exist, each has different priorities which professional and educational organizations should strive to address.

## 1. Introduction

The General Pharmaceutical Council (GPhC, the Great British pharmacy regulatory body) considers a pharmacy professional ‘fit to practise’ when they “can demonstrate the skills, knowledge, character and health required to do their job safely and effectively” [1]. Fitness to practise (FtP) is considered to be “a person’s suitability to be on the register without restrictions” [1]. Similar definitions are provided by other UK healthcare regulators including the General Dental Council (GDC), General Medical Council (GMC) and the Nursing and Midwifery Council (NMC) [2,3,4]. FtP impairment can have a detrimental effect on patient safety and professional reputation. Additionally, for the person undergoing the FtP investigation, subsequent hearings and determination(s) may have a significant impact on their health and career [5,6]. Possible outcomes of an FtP investigation include: no action, a warning (or caution), undertaking, conditions imposed on professional practice, suspension from practising, and removal (erasure or striking off) from the register [1,2,3,4]. Therefore, it is important that educators and professional organizations reflect on published cases to ascertain what lessons can be learnt in an effort to prevent similar issues arising again in the future. Regulatory organizations provide FtP information in their annual reports which is largely summary statistics (particularly medicine and pharmacy) about how the FtP concern was brought to light in the first instance, the number of concerns, and outcomes [7,8,9].

In terms of previous research about FtP cases in the last ten years, Tiffin and colleagues (2017) conducted a national study predicting FtP events in international medical graduates (those registering via the Professional and Linguistic Assessments Board (PLAB)) and ascertained that the PLAB system (specifically, lower scores in parts 1 and 2 and needing to undertake multiple resit attempts) was a valid predictor of eventual censure (including a warning) [10]. Humphrey and colleagues (2011) assessed whether higher impact FtP decisions were more likely to occur for doctors qualified outside the UK than those qualified inside the UK [11]. They found that only 1% of FtP inquiries about UK qualified doctors resulting in erasure or suspension decisions, compared with 4% (for doctors who qualified elsewhere in the European Union (EU)) and 3% (for non-EU qualified doctors). Sanders and Taylor (2013) analyzed the effect of medical school on the incidence of GMC FtP sanctions [12]. They reported that some UK medical schools had lower or higher rates of graduates who received FtP sanctions, but these were not statistically significant. Wakeford and colleagues (2018) investigated the link between performance at UK medical assessments and FtP sanctions and reported that doctors who were sanctioned had performed substantially lower in the medical assessments and that these assessments were valid FtP outcome predictors [13]. Brindley conducted a review of GDC FtP cases between 2012 and 2015 where conditions (restrictions) had been given. The focus of this was to ascertain the GDC’s expectations around continuing professional development and reflection [14]. She reported that out of 56 FtP cases, 15 were asked to produce reflective logs and 38 were asked to submit personal development plans for regular review. Neville examined GDC FtP cases relating to social media [15] and found that out of all published FtP cases, only 2.4% related to social media guideline breaches. These had mainly been carried out by dental nurses with the most frequent complaint being inappropriate Facebook comments. Additionally, Taylor and colleagues (2017) reviewed GDC and GMC FtP hearings in the context of oral and maxillofacial surgery (OMFS) [16] and reported concern that the number of GDC hearings with relevance to OMFS was increasing. Gallagher and colleagues (2015) investigated whether regulators (GMC and GPhC) considered pertinent factors during deliberations into practitioner misconduct and followed relevant guidance when determining which sanction to apply [17,18]. They found this was the case (that the regulatory organization considered relevant factors at all stages of the deliberations and subsequently followed the required guidance about sanctions). Unlike previous work, this current study examined FtP cases via topic analysis and in a broad sense across various healthcare professions (namely, UK dental, medical, nursing and pharmacy cases), allowing insight to be gained at a uni- and mutli-professional level.

Topic analysis facilitates the identification of recurrent themes or topics in text-rich datasets [19] given that analyzing large amounts of text manually can be error-prone and unscalable. Topic analysis methods have been used for biomedical data in recent years, as has the wider concept of machine learning which is an application of artificial intelligence (AI) [20]. In a big data era, with complex datasets arising from numerous data sources, it is important to have awareness of this data analysis technique. For example, it has been used to determine trends in biomedical informatics in journal articles [21], to analyze Twitter data [22,23], to investigate what can be learnt from online health support communities [24] and patients’ Facebook posts [25] and for web document summarization [26]. These examples use topic model(s) rather than word frequencies. Individual words are not analyzed alone since the meaning depends on the context in which the words are used. Instead, groups (or clusters) of words are analyzed. When discussing a particular topic, it is natural to use particular words. For example, when reading about pharmacy, one would not be surprised to find the words ‘drug’, ‘medicine’, ‘prescription’ and ‘dispensing’. In attempting to identify patterns of words that tend to occur in combination with each other, topic models essentially reverse engineer this process. By applying a topic model to a collection of documents or paragraphs, it is possible to get a sense of the essence of the text and the language being used. Documents are represented in terms of their constituent topics, and topics as their constituent words. Examples of topic models include latent Dirichlet allocation (LDA) and non-negative matrix factorization (NMF) [19,27]. O’Callaghan et al. (2015) suggest that NMF may provide more coherent topics particularly when analyzing niche or non-mainstream content, while LDA topics may have higher levels of generality and redundancy [28]. Lee and Seung (1999) discuss NMF in detail in their Nature paper [27]; it involves the construction of a matrix with scores awarded for each topic within the articles, and each topic can be further scored on its use of words [27]. NMF is further explained in the next section of this paper, using FtP examples to solidify theorical concepts.

The aim of the research was to use topic analysis to examine UK FtP dental, medical, nursing and pharmacy cases to ascertain what lessons can be learnt. The objectives were as follows:

- to demonstrate how topic analysis could be employed for examining published FtP cases;
- to apply the NMF model to enable the identification of topics (themes);
- to determine the extent to which the topics affected the four professions.

From the findings of this research, it appears that topic analysis is an appropriate technique for examining published FtP cases as coherent topics were produced, enabling intelligent labelling assignment by the authors. The most frequently identified topic for the dental, medical and nursing professions was in relation to patient care whereas for pharmacy, it was criminal offences. While overlap in the nature of the cases exists for the four professions, each profession has different priorities which their professional and educational organizations should be informed about and strive to address.

## 2. Materials and Methods

### 2.1. Data Collection

FtP cases were sourced from the websites of the four bodies representing each of the professions (i.e., GDC, GMC, GPhC and NMC). For consistency across the four groups, cases from August 2017 to June 2019 were included. This specific time period was largely dictated based on the availability of the pharmacy cases. Only the last twelve months of FtP cases are publicly available on the GPhC website so initial downloads made during August 2018 (covering the time period August 2017 to August 2018) were supplemented with subsequent downloads in June 2019 (covered the time period August 2018 to June 2019). The publicly available data for the other three professions were downloaded in June 2019.

### 2.2. Nature of the Data

The below text provides information about the nature of the data for each profession.

Dental: Cases referred to one of the Practise Committees for a hearing (such as the Professional Conduct, Health, or Professional Performance Committee, depending on the type of case). These cases relate to UK dentists and dental care professionals regulated by the GDC, including clinical dental technicians, dental hygienists, dental nurses, dental technicians, dental therapists, and orthodontic therapists.

Medical: Cases involving UK medical practitioners (regulated by the GMC) that have been referred to the Medical Practitioners Tribunal Service (MPTS) for a hearing.

Nursing: Cases referred to committee substantive hearing (CSH), committee substantive meeting (CSM), committee substantive order review hearing (CSORH) and committee substantive order review meeting (CSORM). These cases relate to nursing professionals regulated by the NMC i.e., UK nurses and midwives, and nursing associates (England).

Pharmacy: Cases heard by the FtP committee (which operates, and makes decisions, independently of the GPhC) where a pharmacy professional’s FtP is alleged to be impaired. These cases relate to pharmacy technicians and pharmacists who are regulated by the GPhC.

### 2.3. Data Pre-Processing

The downloaded PDF files were converted to text files using the R package pdftools (version 1.8) [29]. To prepare these files for analysis, a number of pre-processing steps were applied, namely:

1. Removal of duplicate cases. Where the minutes of two or more hearings related to the same case identification number, the file with the largest size was retained on the basis that it provided the greatest descriptive detail
2. Removal of ‘boilerplate’ text [30] that appears as a standard across many cases. For example, the name of the assembled committee, or the address where the meeting took place
3. Tokenization (i.e., separating text into the constituent words, or ‘tokens’ that comprise the sentences and paragraphs within it) [31]
4. Removal of all tokens not entirely comprised of alphabetic characters (this removed all numeric tokens)
5. Removal of stop words (words that occur with high frequency but add little contextual meaning, for example, ‘the’, ‘and’, ‘but’, ‘in’) [32]
6. Removal of frequently appearing proper nouns including personally identifying names, and place names
7. The conversion of text to lower case

Applying these pre-processing steps was important as they reduce the computational effort required to extract results and hence increase the interpretability of the results (largely by removing words which are unlikely to provide any meaningful context). NB: Words may be filtered based on a pre-defined dictionary [33] but this can raise issues around which dictionary to use. Moreover, given that each profession is likely to include terms and acronyms unique to its vocabulary, and the importance of not eliminating words that could provide insight, a dictionary-filtering approach was not employed in this study.

### 2.4. Topic Extraction

Initially, the FtP dataset was comprised of cases expressed in terms of words from which insights cannot readily be derived. The machine learning topic extraction process seeks to enable understanding by re-expressing the cases as topics which are readily interpretable by humans. The process began by expressing cases in terms of their word frequencies. The information was encoded as a matrix C_W_, comprising of N_C_ (the number of cases) rows and N_W_ (the number of unique words) columns. The NMF process approximately decomposed the matrix C_W_ into the product of two matrices C_T_ (expressing cases in terms of topics) and T_W_ (expressing topics in terms of words) such that C_W_ ≈ C_T_ T_W_. Critically, each of the derived matrices is of lower dimensionality. Matrix C_T_ comprises N_C_ rows and N_T_ (number of topics) columns. The entry at row i, column j reveals a score representing the degree to which topic j features in case i. Matrix T_W_ comprises N_T_ rows and N_W_ columns. The entry at row i, column j reveals a score representing the degree to which word j features in topic i. A key feature of NMF is that all elements of C_T_ and T_W_ are constrained to be non-negative so the presence of one topic is not assumed to reduce the prevalence of particular words. Following O’Callaghan et al. (2015), the approach of log-based term-frequency inverse-document-frequency was adopted [28]. The word frequency for each document (FtP case) was scaled by the inverse of the frequency of documents containing the word. In this way, words which appear frequently in one document but also appear frequently across other documents lose their potency of meaning. Implementation of the model was performed using the scikit-learn Python package (version 0.20.3) [34]. For reference, the model’s df_min and df_max parameters were set at 2% and 50% respectively (i.e., words were only included if they appeared in at least 2% of all documents, and no more than 50%).

### 2.5. Choosing the Number of Topics

Two measures were employed to guide the choice of the number of topics, namely coherence and entropy [28,35,36]. Coherence attempts to measure the meaningfulness of the connections between words within a given topic. Mathematically, this measures the frequency with which pairs of words within a topic co-occur in the same document. High levels of coherence should occur in topics that intuitively make sense to human readers. Entropy attempts to measure information gain; as more topics are added it is anticipated that more information can be gleaned (up to a point). In the case of both coherence and entropy, a reasonable number of topics can be determined by considering the point at which adding more topics fails to provide significant further gain. The limited number of available pharmacy cases and author judgement regarding topic interpretability were also considerations.

### 2.6. Data Analysis

To make sense of the outputs of the NMF process, examination of the two aforementioned matrices was undertaken. Matrix T_W_ represents the topics through a series of scores indicating the prevalence of words. To interpret each topic, the words with the 10 highest scores were examined and manually assigned labels (topic titles) using the authors’ judgement where a coherent theme emerged. Unsurprisingly, many of the words forming topics related to the process of the FtP hearings themselves (such as member, lay, chair, allegation, notified, write, representation); these topics were not included in the analysis. Matrix C_T_ represents cases through a series of scores indicating the prevalence of topics. Thus, a case can relate to several topics with differing degrees of strength. To avoid associating cases to topics with which they were only weakly associated, only topics which had a normalized score above 10% were considered for each case. As a validation check, manual reviews were conducted on the complete pharmacy dataset to confirm that associated topic titles represented the nature of the FtP case.

### 2.7. Ethical Approval

Ethical approval was not sought for this work as the FtP cases under consideration were freely available via the internet (i.e., we only examined publicly available data). Moreover, only anonymized data is presented in this paper. Raw text files of the downloaded cases have not been provided as part of this paper since these are made publicly available by the respective regulatory organization, but for a limited time frame only.

## 3. Results

A total of 3320 FtP cases were included in the analysis.

- 577 dental (as of July 2019, there were around 40,000 dentists and 60,000 dental care professionals on the register [37]).
- 481 medical (as of July 2019, there were around 290,000 UK medical practitioners on the register [38])
- 2199 nursing (as of 31 March 2019, there were 698,237 people on the NMC register [9])
- 63 pharmacy (as of 31 March 2019, there were 56,288 pharmacists and 23,387 technicians on the GPhC register [7]).

The success of the NMF modelling was evident from the fact that coherent topics were produced, enabling intelligent labelling assignment by the authors. Coherence and entropy were ascertained for each dataset (each profession) and for the combined dataset (all professions). In this work, forty topics were chosen for individual profession analysis and one hundred for the simultaneous analysis across all professions.

Table 1 presents the assigned topic titles using example 10-word groupings. Titles were assigned for the individual (each profession) and combined (all professions) datasets. In total, seven topic titles were assigned, namely:

- Criminal offences
- Dishonesty
- Drug possession/supply
- English language
- Indemnity insurance
- Patient care
- Personal behavior

On some occasions, a second title was assigned to the group of words. For example, if there was enough unambiguous information in the word group about what the criminal offence(s) or personal behavior related to, this became the sub-title. Nine sub-titles were assigned, namely:

- Aggression
- Assault
- Competency
- Fraud
- Sexual conduct
- Terrorism-related
- Theft
- Traffic
- Substance misuse

**Figure 1:**
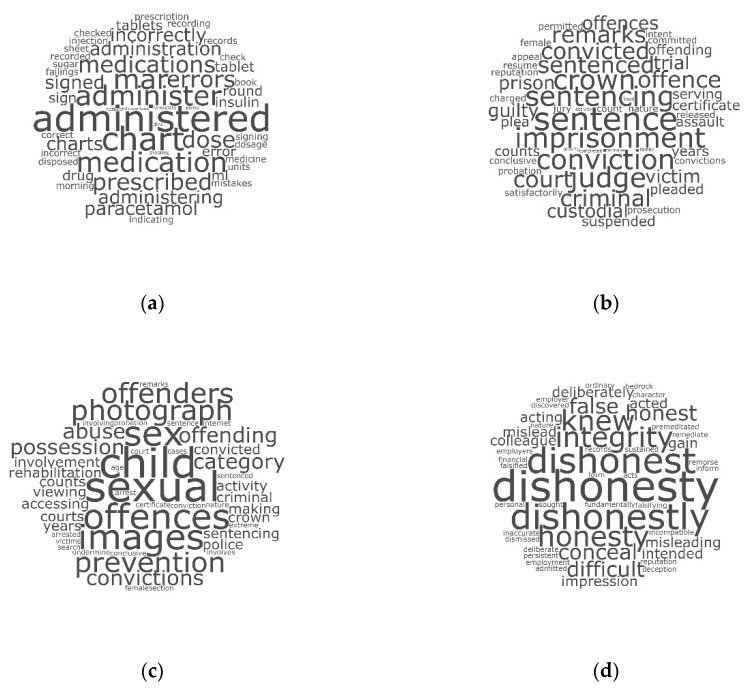
Word clouds depicting the importance of words (font size increases as the word score increases) using the top-50 scoring words within each of the following example topics: (**a**) Patient care (competency); (**b**) Criminal offences; (**c**) Criminal offences (sexual conduct); (**d**) Dishonesty.

In addition, word clouds provide a useful visualization that helps to both confirm and communicate the essence of each topic. By constructing word clouds using the top-50 scoring words per example topic, Figure 1 provides corroborating evidence that the allocated topic titles were appropriate.

Table 2 outlines the results from the combined professions and reveals the proportion of cases associated with each topic. The top result for all professions except pharmacy related to patient care issues including competency. The top result for pharmacy was criminal offences. Pharmacy was the only profession to have 0.00% in relation to English language and personal behavior (aggression). This does not mean that aggressive behavior or English language did not feature in any Pharmacy FtP case; it merely means that neither emerged as a prominent topic (i.e., had a topic score above 10%) in any case.

## 4. Discussion

A comparison of topic prevalence across the four professions revealed that patient care issues were the most frequently identified for three of the four professions (dentistry, medicine and nursing) but pharmacy differed by having the highest proportion relating to criminal offences. The most recent annual dental and nursing reports available (2017 data) showed that the majority of FtP hearings related to ‘failure to provide good quality care’ to patients [9,37], which is similar to our findings. Issues about patient care and competency expose a training need. Similarly, Donaldson et al. (2014) identified poor performance in the UK national medical workforce over an eleven-year period [39]. While teaching and assessment about clinical competency should be embedded at undergraduate level, adequate support also needs to be provided by professional bodies following graduation to ensure this is maintained or enhanced post-qualification, given the dynamic nature of healthcare. This may mean reviewing the provision of continuing professional development opportunities. Research conducted on doctors in New Zealand found that seventy-five percent who entered a 12-month remedial education program (due to clinical performance shortfalls) were subsequently practising at an acceptable standard at the end of remediation [40]. Myers and colleagues (2015) investigated whether doctors remembered new learning in the long-term. They found that doctors’ prescriptions relating to oxygen improved significantly immediately after an educational intervention, but that this improvement was not sustained at a 4-year follow-up [41]. Jayaweera and colleagues (2018) reviewed various assessments to determine whether each contributed something unique to general medical practitioner ‘Tests of Competence’ and ascertain if information could be gleaned about FtP (since these tests are conducted on those referred for FtP issues). They found simulated surgery (SimSurg) had predictive ability in the presence of Objective Structured Clinical Examinations (OSCEs) and the knowledge test in distinguishing doctors from different FtP categories, but that OSCEs did not [42]. Lastly, it should be noted that while lifelong learning and assessment are important, other factors that affect patient care provision include: the organization, its infrastructure and leadership, and resource provision and allocation.

While not the most frequently occurring topic for pharmacy, issues relating to patient care and incompetence still represented a large proportion of the identified topics for this profession. Potentially, there is under-reporting of such issues in an FtP context given the greater autonomy of community pharmacists than the other professions. Alternatively, it could be because they have traditionally held a limited clinical role in comparison to the other healthcare professions and hence have less chance of clinical incompetence or inadequate patient care being exposed. However, as the role of the pharmacist is evolving to be more clinical and less administrative, pharmacists must demonstrate that they have the necessary attributes to undertake these greater responsibilities safely and effectively. Of concern is a recent announcement (news) published by the Pharmacists’ Defence Association (PDA), a UK-based organization that provides assistance to pharmacists about professional indemnity, public liability and statutory liability claims. The news feature states that some doubts have been raised about pharmacists’ competence around diagnosis and prescribing. The PDA concludes by stating that “if pharmacists are expected to assume prescribing and diagnosing responsibilities within primary care, then similar levels of training and support should be provided that have been for doctors” [43].

In addition, English language ability made up a small proportion of identified topics (albeit not to any notable extent for pharmacy). Tiffin and colleagues (2017) found (by examining Professional and Linguistic Assessments Board (PLAB) tests) that international medical graduates working in the UK were more likely to be involved in the FtP process in comparison to home graduates [10]. Donaldson et al. (2014) also found that doctors whose first medical qualification was obtained outside the UK were more likely to have performance issues than UK-qualified doctors [39]. Suggestions to counteract this issue include making assessments such as PLAB tests more stringent, increasing the pass mark, and capping the number of resits permitted. In 2016, the GPhC reported on new legislation whereby all pharmacy professionals (who qualified in the European Economic Area and are seeking to work in Great Britain) must provide evidence that they have the necessary skills in English language to practise safely and effectively [44].

Criminal offence prevalence within pharmacy is concerning of itself, particularly when compared to the other professions, and carries reputational risk. This is something that the professional organization should further investigate. Potentially early interventions and support could help (for example, in relation to substance misuse). While not related to criminal activity per se, a UK-based study involving pharmacists examined the association between job characteristics, well-being and behavior reflecting risky practice (*n* = 517) via a questionnaire study. Two behavioral elements were found, namely “overloading (taking on more work than one can comfortably manage) and risk taking (working at or beyond boundaries of safe practice).” The authors suggest that situational factors should be considered in tandem with personal factors when judging or remediating a person’s FtP [45]. In addition, Paton and colleagues (2018) investigated predictors of FtP declarations among UK medical undergraduates. For conduct-related declarations, predictors included male gender, white ethnicity and a non-professional parental background [46]. Similar research could be conducted in a pharmacy context. Additionally, while UK pharmacy undergraduate students are subject to FtP regulations, subsequent FtP cases are not openly published by the universities (unlike the regulator) which means opportunities to interrogate the data and reflect on the key findings are limited.

The high proportion of drug possession and/or supply cases within pharmacy is also noteworthy. Perhaps this is opportunistic or related to the level of interest and expertise in the subject area. Likewise, inappropriate sexual conduct appeared to be most prevalent for practitioners who have to physically examine patients (doctors), although this does not explain why it was lowest among the nursing profession.

### Limitations

The first limitation is the availability of the datasets. As previously mentioned, while regulatory organizations make FtP cases publicly available on their websites, they restrict the time frame that these are available for (this is understandable given the potential impact on those involved). Moreover, the pharmacy dataset (number of cases) was much smaller than the other professions’ datasets which means the findings should be interpreted with caution. Furthermore, a difficulty in topic analysis is the requirement to specify the number of topics. Choosing too few may lead to overly broad topics which cannot easily be classified, however, choosing too many may lead to overly narrow, spurious or infrequently discussed topics, from which no generalizable insights can be drawn.

## 5. Conclusions

This paper has shown how UK FtP cases (from dental, medical, nursing and pharmacy professions, *n* = 3320 in total) were interrogated using NMF topic model analysis. It appears that topic analysis was an appropriate technique for examining such cases and the success of the NMF modelling was evident as coherent topics were produced, enabling intelligent labelling assignment by the authors. This approach could be considered for other pharmacy research involving interrogation of other text-rich documents such as medicine information leaflets.

Identified FtP topics among all professions combined were criminal offences, dishonesty (including fraud), drug possession/supply, patient care (including incompetence) and personal behavior (including sexual conduct). The most frequently identified topic for the dental, medical and nursing professions was in relation to patient care, whereas for pharmacy it was criminal offences. While overlap exists across the professions, each has different priorities which their professional and educational organizations should be informed about and strive to address.

## Figures and Tables

**Table 1:** Allocated topic titles and sub-titles (illustrated using examples of the top 10 scoring words).

Groups of Words (10 Highest Scoring Words)	Allocated Topic Title (Sub-Title)
sentence, sentencing, conviction, crown, imprisonment, judge, sentenced, convicted, court, remarks	Criminal offences
assault, conviction, violence, beating, magistrates, police, criminal, court, guilty, assaulting	Criminal offences (assault)
sexual, child, images, sex, offences, offenders, photograph, prevention, convictions, photographs	Criminal offences (sexual conduct)
jury, terrorism, conviction, Islamic, murder, trial, prison, judge, sentencing, defendant	Criminal offences (terrorism-related)
speeding, traffic, offences, driver, vehicle, drivers, liable, declarations, magistrates, sentences	Criminal offences (traffic)
dishonesty, dishonest, dishonestly, honesty, integrity, knew, conceal, false, difficult, honest	Dishonesty
falsified, forged, signatures, signature, false, submitting, purported, verification, dishonest, stamp	Dishonesty (fraud)
cash, thefts, money, till, theft, planned, additionally, caution, repay, repaid	Dishonesty (theft)
drugs, controlled, drug, misuse, possession, supply, class, book, theft, quantity	Drug possession/supply
English, language, registrar, knowledge, kingdom, united, qualification, speaking, score, skills	English language
indemnity, insurance, cover, compensation, indemnified, hold, arrangements, claim, membership, policy	Indemnity insurance
gloves, control, instruments, infection, decontamination, cross, nurses, items, cleaned, inspection	Patient care
administered, chart, administer, mar, medication, prescribed, dose, errors, medications, incorrectly	Patient care (competency)
factors, attitudinal, behavior, harm, deep, seated, mark, harmful, personality, actions	Personal behavior
room, words, link, video, conversation, nurse, aggressive, staff, call, rude	Personal behavior (aggression)
sexual, touching, breasts, boundaries, sexually, touched, harassment, thigh, leg, knee	Personal behavior (sexual conduct)
cannabis, cocaine, consumed, abstinence, coping, mid, relapse, redacted, results, hair	Personal behavior (substance misuse)

**Table 2:** Topics extracted from the combined professions (*n* = 100, normalized score >10%) and proportion of cases associated with each topic.

Topics	Dental	Medical	Nursing	Pharmacy
Criminal offences	16.8%	17.9%	6.3%	38.1%
Criminal offences (sexual conduct)	1.4%	3.5%	1.5%	3.2%
Criminal offences (substance misuse)	3.1%	3.3%	1.0%	4.8%
Dishonesty	0.7%	0.4%	0.8%	4.8%
Dishonesty (fraud)	8.7%	3.3%	2.0%	14.3%
Drug possession/supply	1.6%	1.7%	2.2%	28.6%
English Language	1.2%	2.5%	3.0%	0.0%
Indemnity Insurance	6.2%	0.8%	0.0%	0.0%
Patient Care	25.5%	25.6%	8.6%	6.3%
Patient Care (competency)	4.0%	38.9%	17.9%	17.5%
Personal behavior	0.0%	0.0%	0.8%	0.0%
Personal behavior (aggression)	0.3%	1.5%	3.3%	0.0%
Personal behavior (sexual conduct)	0.9%	5.6%	0.6%	3.2%
